# Cinnarizine for the prophylaxis of migraine associated vertigo: a retrospective study

**DOI:** 10.1186/2193-1801-3-231

**Published:** 2014-05-07

**Authors:** Foad Taghdiri, Mansoureh Togha, Soodeh Razeghi Jahromi, Farshid Refaeian

**Affiliations:** Iranian Center of Neurological Research, Neuroscience Institute, Tehran University of Medical Sciences, Tehran, Iran; Department of Neurology, Neurology Ward, Sina Hospital, Tehran University of Medical Sciences, Imam Khomeini St, 11367-46911 Tehran, Islamic Republic of Iran; Multiple Sclerosis Research Center, Neuroscience Institute, Tehran University of Medical Sciences, Tehran, Iran

**Keywords:** Cinnarizine, Vertigo, Vestibular migraine, Migraine associated vertigo, Basilar migraine, Brainstem migraine

## Abstract

**Objective:**

To assess the efficacy and safety of cinnarizine for the prophylaxis of migraine associated vertigo in the vestibular migraine and migraine with brainstem aura.

**Background:**

Vestibular migraine and migraine with brainstem aura are two principal clinical syndromes that frequently are associated with vertigo. Since cinnarizine is a well-tolerated calcium channel blocker which has acceptable effect on both vertigo and migraine headache, we carried out this study to evaluate the efficacy and safety of this medication in vestibular migraine and also migraine with brainstem aura associated with vertigo.

**Methods:**

This was a retrospective, single-center, open-label, investigation of the effects of cinnarizine on vestibular migraine and migraine with associated with vertigo. We assessed the change in monthly frequency of vertigo and also frequency, duration and intensity of migraine attacks after one, two and three months of cinnarizine administration.

**Results:**

The mean frequency of vertigo and also the mean frequency, duration and intensity of migraine headaches per month were reduced significantly after three months of cinnarizine therapy (all p < 0.001).

**Conclusion:**

This study suggests that cinnarizine is safe and effective in reducing both headache and vertigo aspects of “migraine plus vertigo” among the patients who suffer from either vestibular migraine or migraine with brainstem aura associated with vertigo.

## Background

Migraine and vertigo are two of the most common disorders in the general population that they tend to occur together (Mehmet 2011). Recent studies suggest that the comorbidity is about 3.2% and up to 25% of patients with migraine may experience vertigo (Neuhauser 2009). Meanwhile, migraine has been considered as one of the most frequent disorders that causes vertigo according to various epidemiological studies (Riina et al. 2005; Vuković et al. 2007). Vestibular migraine (VM) (or migrainous vertigo) and migraine with brainstem aura (BM) (previously known as basilar-type migraine) are two principal clinical syndromes that are associated with vertigo (Neuhauser 2009; Brandt 2004). The International Headache Society (IHS) has accepted both VM and BM as migraine variants and has indicated that only a minority of VM patients experience vertigo in the time frame of 5–60 minutes that is defined for an aura symptom. On the other hand, IHS requires an aura with two or more brainstem aura symptoms in addition to visual, sensory or dysphasic aura symptoms to qualify as BM criteria. Thus, these variants should be considered as two different migraine types (Society HCCotIH 2013).

Several studies suggest that medications used for migraine prophylaxis may also be effective in comorbidity of migraine and vertigo (Reploeg and Goebel 2002; Waterston 2004; Dieterich and Brandt 1999). Cinnarizine (CIN) is a well-tolerated L-type calcium channel blocker which has early onset effects on migraine prophylaxis (Togha et al. 2006; Mansoureh et al. 2008). While, CIN directly inhibits vestibular hair cells stimulation (Arab et al. 2004) and also has antihistaminic actions (Pianese et al. 2002), it seems to be a good choice in treatment of migraine with vertigo. However, to the best of our knowledge, no investigation has yet been made to assess the efficacy and safety of CIN in the treatment of migraine and vertigo comorbidities. Therefore, we carried out this retrospective study to evaluate the efficacy and tolerability of CIN in vestibular migraine and also migraine with brainstem aura associated with vertigo.

## Methods and patients

This was a retrospective, single-center, open-label, investigation of the effects of cinnarizine (CIN) on migraine associated vertigo in vestibular migraine (VM) and migraine with brainstem aura (BM) that conducted in the headache clinic of Neurology Department, Tehran University of Medical Sciences with both physician- and self-referred patients. The study protocol was approved by the ethics committee of Tehran University of Medical Sciences.

### Inclusion and exclusion criteria

Vestibular migraine (VM) and migraine with brainstem aura (BM) were defined according to the criteria of the Headache Classification Committee of the International Headache Society (IHS) 3^rd^ edition (beta version) (Lempert et al. 2012; Society HCCotIH 2013). Additional inclusion criteria that specified patients were as follows: men and women, age between 18 and 60 years, reporting of vertigo in more than 50% of attacks, a history of migraine for at least one year, onset of migraine before the age of 50. Patients were included in the study group if they met all these criteria and also had at least one return visit after CIN was prescribed and if the patients stated that they had consistently taken the medication. At the time of the prescription neither the patients nor the physician knew of the investigation.

### Study design

Patients complaining vertigo regardless of vertigo attacks time (during the headaches or between the headache attacks) who met all entry criteria were categorized into two different groups of VM-group and BM-group. All of the data were collected from the patients’ headache diaries in which all vertigo attacks, migraine attacks, duration of attacks (hours), and intensity of attacks (assessed by a 10-score Visual Analog Scale (VAS), which “0” indicating no pain and “10” the worst pain imaginable) were recorded. Cinnarizine treatment regimen was initiated by a 37.5-mg tablet (Cinnageron Tablets, G. Streuli & Co. AG, Uznach) at bedtime for the first three days and then 75-mg tablet at bedtime for the remaining treatment period. Office follow-up visits were performed one, two and three months after the CIN treatment initiation and the change in monthly frequency of vertigo attacks and migraine attacks during the study were considered as the main and primary outcome, while secondary variables were duration of migraine attacks in hours and intensity of the attacks (VAS).

From the first visit on, patients were asked if they had experienced any adverse events. Although we recorded all the adverse events, special attention was paid to occurrence of weight gain and extra pyramidal symptoms.

### Statistical analysis

Data are expressed as mean ± standard deviation unless otherwise stated. For intergroup continuous variables comparisons One-way ANOVA or Kruskal-Wallis tests were used. For post hoc Turkey HSD or Bonferroni adjusted Mann–Whitney test was used. For within group comparisons of continuous variables, paired sample student t-test or Wilcoxon sign test was applied. For binomial variables, Fisher’s exact tests or Pearson’s chi-square tests were done for between-group comparisons. Two-sided p value less than 0.05 considered as statistically significant for all tests. Data were analyzed using SPSS Statistics version 20. (SPSS Inc., Chicago, IL, USA).

## Results

The study included twenty-four subjects with VM (23 women, 1 man) and sixteen subjects with BM (12 women, 4 men). The ages of subjects ranged from 18 to 54 years (mean, 30.0 years). Two (5%) of the 40 subjects were lost to follow up and did not return after the two months on CIN. None of the remaining patients discontinued the CIN treatment due to adverse events or any other causes. Demographic and baseline headache characteristics were similar except for the mean attacks duration which was higher in the BM-group (Table 1).

**Table 1:** **Demographics and baseline characteristics**

Variable	Vestibular migraine (n = 24)	Migraine with brainstem aura (n = 16)	p-value
Age, years (mean ± SD)	31.2 ± 8.0	28.4 ± 11.0	0.084
Duration of migraine (mean ± SD)	7.23 ± 7.2	7.94 ± 4.8	0.291
Baseline monthly frequency of migraine attacks (mean ± SD)	3.92 ± 0.93	4.19 ± 1.5	0.845
Baseline monthly duration of migraine attacks, hours (mean ± SD)	23.6 ± 15.8	30.1 ± 14.1	0.011
Baseline monthly intensity of migraine attacks, VAS (median (range))	8 (7–10)	9 (7–10)	0.087

SD = standard deviation.

### Patients with vertigo (total vertigo population)

Since, vertigo was a common symptom in our all 40 subjects; so we first assessed the efficacy of CIN in migraine with vertigo regardless of patients’ migraine type. The overall reduction in the frequency, duration and intensity of the migraine attacks was substantial. The mean frequency and duration of migraine headaches per month were reduced from 4.02 ± 1.2 and 26.20 ± 15.3 hours at baseline to 1.10 ± 0.9, 4.18 ± 3.6 hours at the final visit, respectively (all p < 0.001). The median and range of headaches’ intensity (VAS) were also decreased from 8 (7–10) at baseline to 3 (0–8) at the final visit (p < 0.001). Improvement was noted in most patients as early as at one month compared with baseline (Table 2).

**Table 2:** **Headache characteristics before and after treatment with cinnarizine**

Variable	Total vertigo population (n = 40)	Subgroups		
		Vestibular migraine (n = 24)	Migraine with brainstem aura (n = 16)	p-value^1^
**Monthly frequency of migraine attacks (mean ± SD)**				
At baseline	4.02 ± 1.2	3.92 ± 0.9	4.19 ± 1.5	0.845
12-week treatment period				
1^st^ 4-week	2.35 ± 1.1*	1.92 ± 0.6*	3.00 ± 1.3*	0.001
2^nd^ 4-week	1.30 ± 1.2*	0.71 ± 0.8*	2.19 ± 1.0*	<0.001
3^rd^ 4-week	1.10 ± 0.9*	0.75 ± 0.7*	1.62 ± 1.0*	0.004
**Monthly duration of migraine attacks, hours (mean ± SD)**				
At baseline	26.20 ± 15.3	23.58 ± 15.8	30.12 ± 14.1	0.011
12-week treatment period				
1^st^ 4-week	14.78 ± 6.3*	13.88 ± 7.0*	16.12 ± 4.9*	0.066
2^nd^ 4-week	5.68 ± 5.1*	3.46 ± 4.1*	9.00 ± 4.6*	0.001
3^rd^ 4-week	4.18 ± 3.6*	2.58 ± 3.0*	6.56 ± 3.2*	<0.001
**Monthly intensity of migraine attacks, VAS (median, Max.-Min.)**				
At baseline	8 (7–10)	8 (7–10)	9 (7–10)	0.087
12-week treatment period				
1^st^ 4-week	6 (4–10)*	5 (4–9)*	7 (5–10)*	0.005
2^nd^ 4-week	3 (0–9)*	1 (0–7)*	5 (2–9)*	<0.001
3^rd^ 4-week	3 (0–8)*	1 (0–5)*	4 (2–8)*	<0.001

*VAS* Visual Analog Scale.

*p < 0.05 compared with baseline.

^1^p-value for comparison between vestibular migraine and migraine with brainstem aura.

### Vestibular migraine

In the VM-group, the mean frequency of migraine headaches per month decreased from 3.92 ± 0.9 before starting the medication to 0.75 ± 0.7 (p < 0.001) at the last visit. The mean duration and median intensity of migraine headaches per month were also reduced from 23.58 ± 15.8 hours and 8 (min: 7 - max: 10) at baseline to 2.58 ± 3.0 hours and 1 (min: 0 – max: 5) at the final visit, respectively (p < 0.001). The reductions in all three efficacy variables were significant at every time point after one month. The greatest reduction in frequency and duration of attacks was seen during the second month of treatment, with the frequency reduced to 0.71 ± 0.8 migraine headaches per month, and the average duration of migraine headaches dropped to 3.46 ± 4.1 hours per month (p < 0.001) (Table 2).

### Migraine with brainstem aura

In the BM-group, the mean frequency of migraine headaches per month decreased from 4.19 ± 1.5 before starting the cinnarizine to 1.62 ± 1.0 (p < 0.001) at the last visit. The duration and median intensity of migraine headaches per month were also reduced from 30.12 ± 14.1 hours and 9 (min: 7-max: 10) at baseline to 6.56 ± 3.2 hours and 4 (min: 2-max: 8) at the final visit, respectively (p < 0.001). The reductions in all three efficacy variables were significant at every time point after one month (Table 2).

### Comparison between the two migraine types

Although CIN has significantly improved efficacy variables in both VM- and BM-groups, reduction in these variables was significantly greater in VM-group. Except for baseline monthly duration of migraine attacks, all other demographic and baseline headache characteristics were similar in groups. As shown in Table 2, patients in VM-group showed greater decreases in mean frequency and intensity of migraine attacks per month compared with the patients who fulfilled the criteria for migraine with brainstem aura (p < 0.01). Although the mean duration of migraine headaches per month was significantly shorter in the VM-group at the baseline, it becomes similar in both groups after one month (p = 0.066) and again after the second and third months, greater reduction was seen in the mean duration of monthly migraine headaches among the patients with VM.

### Vertigo

In the BM-group and VM-group, the mean frequency of vertigo per month decreased from 3.50 ± 0.89 and 3.79 ± 1.14 before starting the cinnarizine to 1.62 ± 1.0 and 0.42 ± 0.65 at the last visit, respectively (p < 0.001). Meanwhile, the reductions of vertigo frequency were significant at every time point after one month (Table 3). Although significant reductions in monthly frequency of vertigo were seen in both subgroups, the reductions were not significantly different when compared between the subgroups.

**Table 3:** **Vertigo frequency before and after treatment with cinnarizine**

Variable, mean ± SD	Total vertigo population (n = 40)	Subgroups		
		Vestibular migraine (n = 24)	Migraine with brainstem aura (n = 16)	p-value^1^
**Monthly frequency of vertigo attacks**				
At baseline	3.68 ± 1.05	3.79 ± 1.14	3.50 ± 0.89	0.372
12-week treatment period				
1^st^ 4-week	1.30 ± 1.07*	1.54 ± 1.10*	0.94 ± 0.93*	0.079
2^nd^ 4-week	0.60 ± 0.67*	0.79 ± 0.72*	0.31 ± 0.48*	0.016
3^rd^ 4-week	0.35 ± 0.65*	0.42 ± 0.65*	0.25 ± 0.58*	0.402

*p < 0.05 compared with baseline.

^1^p-value for comparison between vestibular migraine and migraine with brainstem aura.

Overall 9 subjects (22.5%) reported adverse events during the study period. 5 subjects (12.5%) experienced weight gain, 3 subjects (7.5%) reported blurred vision, and one patient (2.5%) reported somnolence. Neither of our subjects experienced parkinsonism during the study.

## Discussion

Our experience suggests that CIN is safe and effective in reducing the frequency of vertigo attacks as well as frequency, duration and intensity of migraine headaches among the patients who suffer from either vestibular migraine or migraine with brainstem aura associated with vertigo. Although, CIN worked effectively with both types of migraine, reducing the headache characteristics (frequency, duration and intensity) by a statistically significant level in the first month of treatment, the medication was more effective in vestibular migraine subtype. On the other hand, the frequency of vertigo attacks was not reduced differently between subtypes that this finding may reveal various aspects of CIN in migraine treatment and can be considered that CIN has independent effects on vertigo and headache characteristics of “migraine plus vertigo”. However, these findings should be confirmed by other studies.

Cinnarizine is a selective calcium channel blocker that has been used to prevent and treat vertigo. Several studies reported extrapyramidal reactions and depression that were induced by CIN. However, these adverse events are usually described in elderly patients with prolonged CIN therapy (Negrotti and Calzetti 1997; Martí‒Massó and Poza 1998; Micheli et al. 1987). Overall 22.5% of our subjects reported adverse events with CIN, which all were mild to moderate, and no serious side effects such as extrapyramidal reactions or depression were reported during the medication administration. Meanwhile, some previous studies have also shown that this medication can be well tolerated in patients suffering from migraine (Mansoureh et al. 2008; Togha et al. 2006).

In two different recent studies, authors concluded that cinnarizine, either alone or in combination with dimenhydrinate is an effective and well-tolerated option for treatment of vertigo with various origins (Scholtz et al. 2012; Hahn et al. 2011).

Some studies reveal that prophylactic treatments for migraine such as ergots and beta-blockers are efficient in preventing migraine related vertigo (Tusa 2000). But, these drugs have limitations to use in asthmatic and cardiovascular diseases. It is difficult to interpret most of these studies, because they assessed different disease entities, and thus, cannot be compared directly.

However, in a retrospective study, Baier et al. compared the treatment response of VM in a group of patients who received prophylactic medications such as beta-blockers, valproic acid, topiramate, lamotrigine, amitriptyline and flunarizine, with another group who did not receive any prophylactic medications, and reported that patients with medical prophylaxis experienced a reduction in frequency (80%), intensity (68%) and duration (65%) of the episodic vertigo attacks (Baier et al. 2009). In another study which investigated the efficacy of lamotrigin in migraine related vertigo, the authors reported that the mean vertigo frequency per month was significantly decreased from 8.1 to 5.4, while the change in mean headache frequency per month was not significant (Bisdorff 2004). In another retrospective study (Bikhazi et al. 1997) authors reported that only little improvement was seen in either headache or dizziness in the 22 patients taking calcium-channel blockers, beta-blockers, antidepressants, valproic acid, methylsergide and cyproheptadine. In contrast, Reploeg et al. reported a dramatic amelioration (72% of the 81 patients) of vertigo attacks with tricyclic antidepressants or beta-blockers in 81 patients with nonspecific migraine-associated vertigo (Reploeg and Goebel 2002).

## Conclusions

However, although our study was small and studies with larger sample sizes and longer follow-up durations are needed to assess the efficacy and safety of CIN in migraine related vertigo, the results of this study are robust and show that CIN may be considered as an excellent choice for treatment of migraine and vertigo comorbidities and particularly vestibular migraine.
